# Metabolite proving fungal cleavage of the aromatic core part of a fluoroquinolone antibiotic

**DOI:** 10.1186/2191-0855-2-3

**Published:** 2012-01-03

**Authors:** Heinz-Georg Wetzstein, Josef Schneider, Wolfgang Karl

**Affiliations:** 1 Bayer Animal Health GmbH, Leverkusen, Germany; 2 Bayer Cropscience AG, Monheim am Rhein, Germany; 3 Currenta GmbH & Co. OHG, Leverkusen, Germany

**Keywords:** fluoroquinolone, pradofloxacin, fungal degradation, *Gloeophyllum striatum*, aromatic ring cleavage, metabolites

## Abstract

Liquid cultures of the basidiomycetous fungus *Gloeophyllum striatum *were employed to study the biodegradation of pradofloxacin, a new veterinary fluoroquinolone antibiotic carrying a CN group at position C-8. After 16 days of incubation, metabolites were purified by micro-preparative high-performance liquid chromatography. Four metabolites could be identified by co-chromatography with chemically synthesized standards. The chemical structures of three compounds were resolved by ^1^H-nuclear magnetic resonance spectroscopy plus infrared spectroscopy in one case. All metabolites were confirmed by high resolution mass spectrometry-derived molecular formulae. They comprised compounds in which the carboxyl group or the fluorine atom had been exchanged for a hydroxyl group. Furthermore, replacement of the CN group and the intact amine moiety by a hydroxyl group as well as degradation of the amine substituent were observed. The chemical structure of a catechol-type fluoroquinolone metabolite (F-5) could be fully defined for the first time. The latter initiated a hypothetical degradation sequence providing a unique metabolite, F-13, which consisted of the cyclopropyl-substituted pyridone ring still carrying C-7 and C-8 of pradofloxacin, now linked by a double bond and substituted by a hydroxyl and the CN group, respectively. Most likely, all reactions were hydroxyl radical-driven. Metabolite F-13 proves fungal cleavage of the aromatic fluoroquinolone core for the first time. Hence, two decades after the emergence of the notion of the non-biodegradability of fluoroquinolones, fungal degradation of all key structural elements has been proven.

## Introduction

Pradofloxacin (PRA), a new fluoroquinolone (FQ) drug, is used to treat bacterial infections in cats and dogs Litster et al. 2007;Mueller and Stephan 2007. It shares the core structure of common cyclopropyl-type FQs Domagala and Hagen 2003 but carries a cyano group at position C-8 and a bi-cyclic amine at C-7, S,S-pyrrolidinopiperidine ([1S,6S]-2,8-diazabicyclo[4.3.0]non-8-yl); the latter is also contained in moxifloxacin Petersen 2006. Concerning *in vitro *antibacterial activity, particularly low mutant prevention concentrations of PRA suggest a high potential for preventing the emergence of resistance under therapy Wetzstein 2005. Both substituents in combination are essential for its improved efficacy Wetzstein and Hallenbach 2011. Furthermore, the CN group facilitates hydrolytic elimination of the amine moiety (i.e., drug inactivation) under the slightly alkaline conditions present in decaying animal waste Wetzstein et al. 2009. Hence, PRA should be more readily biodegradable and thus ecologically favorable than conventional FQs.

Fungal degradation of FQs such as ciprofloxacin Wetzstein et al. 1999, enrofloxacin Karl et al. 2006 and moxifloxacin Petersen 2006 under defined *in vitro *conditions has been described in detail. However, all findings yet reported concerned decomposition of the amine substituent located at C-7 and the pyridone part of the FQ core. Eighty-seven metabolites of enrofloxacin produced by *Gloeophyllum striatum *DSM 9592, a basidiomycetous fungus causing brown rot decay of wood by employing a Fenton-type reaction mechanism Jensen et al. 2001;Qui and Jellison 2004, could be resolved: The chemical structures of 18 metabolites were fully elucidated, while 69 metabolites needed to be postulated based on their molecular formula, retention time and the most likely chemical context Karl et al. 2006. Forty-eight additional metabolites were generated by seven taxonomically diverse *Basidiomycetes *indigenous to agricultural sites, including *Agrocybe praecox *DSM 13167 from soil Wetzstein et al. 2006. Two postulated *cis,cis*-muconic acid-type congeners of enrofloxacin, metabolites 78 and 79 in Karl et al. (2006), came closest to implying aromatic ring cleavage to have occurred. However, although chemically more stable, the latter metabolites may, alternatively, be formulated as tetra-hydroxylated constitutional isomers. Hence, stringent evidence proving biodegradation of the aromatic core part of a FQ has not yet been reported. Our aim was to characterize the basic degradation scheme for PRA in *G. striatum *and to attempt the identification of new types of metabolites, to be expected due to the presence of the cyano group at position C-8.

## Materials and methods

### Isolation and identification of metabolites

Mycelia of *G. striatum *DSM 9592 were grown in a fourfold diluted malt broth, washed and then re-suspended in a defined mineral medium to give a concentration of about 2 mg dry weight per mL, as has been described previously Wetzstein et al. 1997. The culture vessels, screw-capped 250-mL Duran^® ^glass bottles, contained 30 mL of mycelial suspension but were otherwise identical with those described before. PRA was added at 27 ± 0.5 μg/mL, labeled with either 1.4 or 1.9 MBq of [2-^14^C]PRA or [pyrrolidinopiperidine-7-^14^C]PRA, respectively Wetzstein et al. 2009. Both compounds had a radiochemical purity of > 98%. Cultures were then incubated at room temperature in the dark. ^14^CO_2 _produced was quantified as described before Wetzstein et al. 1997.

Emerging metabolites were monitored by high-performance liquid chromatography (HPLC), as described elsewhere Wetzstein et al. 1997. The former eluent system was slightly modified in that the aqueous component A contained 10 mM ammonium formate, 1% formic acid and 1% isopropanol; once again, acetonitrile served as component B. By adding B, component A was linearly decreased to 94% between 2 and 5 min, then to 85% over 9 min, to 70% over 15 min, to 50% over 5 min, and to 0% over 10 min. A shallower gradient (B) needed to be applied during co-chromatographic identification of the metabolites as well as micro-preparative purification of single metabolites from collected peak fractions. The HPLC gradient was modified as follows: By adding compound B, component A was linearly decreased to 94% over 5 min, to 88% over 10 min, to 82% over 15 min, to 75% over 15 min, and to 50% over 5 min. In order to identify metabolite F-10, [pyrrolidinopiperidine-7-^14^C]PRA needed to be provided as substrate. In case of F-10, component B consisted of 10% of acetonitrile in methanol (vol/vol). The flow rate was 1 mL/min, throughout.

Mycelia were separated by centrifugation and the resulting pellets washed with 50 mL of sterile water. The combined supernatants were passed through a 0.45 μm pore-size filter, then freeze-dried and re-suspended in 2 mL of distilled water. Metabolites could be isolated from such stock solutions by micro-preparative HPLC and manual collection of the relevant gradient fractions. After checking purity by HPLC analysis, metabolites were concentrated again by freeze-drying and re-suspended appropriately for structure determination.

### Structure determination

HPLC-mass spectrometry (HPLC-MS) was performed as described previously Wetzstein et al. 1997. To characterize F-10, an isocratic mobile phase was used consisting of equal volumes of 25 mM ammonium acetate in water and methanol. Proton nuclear magnetic resonance (^1^H-NMR) spectra were recorded at 500 MHz in D_2_O containing 5% (vol/vol) of trifluoroacetic acid-d_1_, with acetone serving as an internal standard set at δ = 2.16 ppm Wetzstein et al. 1997. High-resolution mass spectrometry (HR-MS) and determination of the molecular formula have been described by Karl et al. (2006). Fourier transformed infrared spectroscopy (FT-IR) specifically indicated the presence of the conjugated nitrile group in PRA by absorbance at 2205 cm^-1^. Of metabolite F-13, a sample of about 10 μg was imbedded in a micro potassium bromide disc. This was analyzed by using a UMA 500 microscope mounted to a FTS 60A spectrometer (Bio-Rad, Krefeld, Germany).

## Results

### Metabolites of PRA formed by *G. striatum*

After 8 weeks, ^14^CO_2 _production from [2-^14^C]PRA and [pyrrolidinopiperidine-7-^14^C]PRA, applied at 10 μg/mL, had reached 34.3% ± 2.7% and 6.3% ± 1.0% of the initially applied ^14^C-label (average ± SD for three cultures). The kinetics were almost identical to those described previously for [2-^14^C]enrofloxacin and [piperazine-2,3-^14^C]enrofloxacin, respectively. Hence, the data are not shown in detail, but see Figure 2 in Wetzstein et al. (1997).

**Figure 1:**
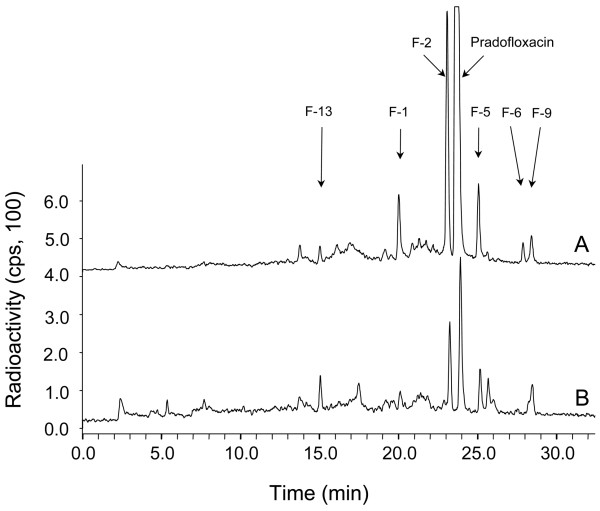
**HPLC elution profiles of concentrated supernatants from cultures of *G. striatum *metabolizing [2-^14^C]PRA after 16 (A) and 42 days (B) of incubation**. In trace (B), the sensitivity of detection was increased threefold.

**Figure 2:**
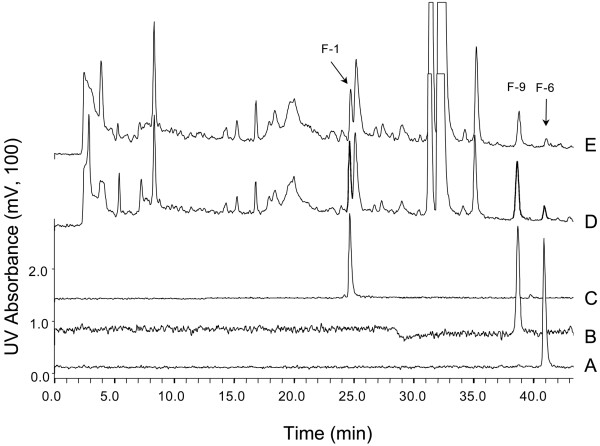
**Co-chromatographic identification of PRA metabolites**. Elution profiles of chemically synthesized references are shown in traces (A): 8-OH-PRA, F-6; (B): the 7-amino metabolite of PRA, F-9; and (C): the 3-hydroxy-congener of PRA, F-1. Trace (E) represents the elution profile of concentrated supernatants. In trace (D), concentrated supernatant had been spiked by adding compounds A, B and C to approximately double their concentrations. Note the reversed order of F-6 and F-9 (as compared to Figure 1), due to the shallower gradient applied; retention times are summarized in Table 1. Absorbance was recorded at 270 nm.

**Figure 3:**
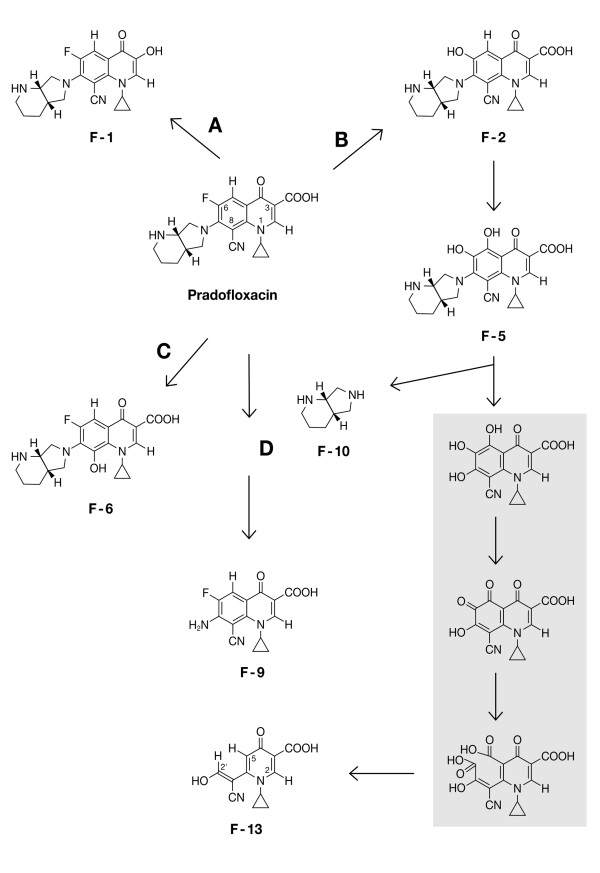
**Metabolic scheme for PRA in *G. striatum***. Degradation routes were initiated by oxidative decarboxylation (A), defluorination (B), hydroxylation of position C-8 (C), and decomposition of the amine moiety (D) which, alternatively, could be eliminated intact as F-10. Hypothetical intermediates are marked in grey: twofold oxidation of the pyrogallol-type intermediate is likely to provide a *cis,cis*-muconic acid-type metabolite, from which F-13 may be formed after two spontaneous decarboxylations.

Chemically synthesized reference standards could be employed to identify F-1, F-6 and F-9 by co-chromatography (Figure 2). F-1 and F-6 were identical with congeners of PRA, mono-hydroxylated at C-3 or C-8, respectively. Hydroxyl radical-driven elimination of the CN group, providing metabolite F-6, is notable. F-9 indicated complete degradation of the amine moiety with the C-7 amino group remaining attached to the FQ core part (Figure 3). Following isolation of F-1, F-6 and F-9 by micro-preparative HPLC, their structures were confirmed by HR-MS-derived molecular formulae and ^1^H-NMR analysis as well (not shown). Molecular weights, retention times and characteristics of the absorption spectra of all metabolites are compiled in Table 1.

HPLC analysis of 16-day-old supernatant revealed the pattern of PRA metabolites produced (Figure 1). Based on previous findings, six major metabolites were designated F-1, F-2, F-5, F-6, F-9 and F-13; F denotes a type of fungal metabolite with proven chemical structure. Their concentrations amounted to 9% (F-2), 2 to 3% (F-1, F-5), and about 1% (F-6, F-9, F-13) of the ^14^C-label applied. Upon prolonged incubation (e.g., after 42 days), the concentrations of F-1, F-2, F-6 and F-5 (three mono and one dihydroxylated congeners, respectively; see below) declined extensively, while those of F-9 and F-13 remained essentially constant. At that point, even PRA had reached the concentration level typical of major metabolites. Hence, supernatants of four parallel cultures were harvested on day 16. The ^14^C-label recovered in the combined supernatants amounted to 92% of the activity applied.

**Table 1:** HPLC retention times, pseudomolecular ions in HPLC-MS, and characteristics of the UV absorption spectra of [^14^C]PRA and its metabolites

Characteristic			Values for					
	
	PRA	F-1	F-2	F-5	F-6	F-9	F-10*^b^*	F-13
Retention time (min)								
Method A	23.8	20.2	23.3	25.3	28.1	28.6	~2.4	15.2
Method B	32.0	24.7	31.4	35.2	41.0	38.7	~2.4	16.8
Pseudomolecular ion								
[M+H]^+ ^(*m/z*)*^a^*	397	369	395	411	388	288	127	247
UV absorption (nm)								
λ_max _(major)	288	250	288	298	242	272	-	280
λ_max _(minor)	364 248	278 362 374	376 392	260 374	298 330	328 350	-	320 362
Shoulder	374 340		260					

*a *[M+H]^+ ^was accompanied by [M+2+H]^+^, resulting from the ^14^C-label;

*b *Data obtained in an experiment with [pyrrolidinopiperidine-7-^14^C]PRA

Due to their instability, no reference compounds were available of F-2 and F-5. However, the NMR spectrum of F-2 showed two singlets (Table 2): one, assigned to the proton at position C-5 (δ = 7.64 ppm), proved the absence of fluorine, while the other was due to the proton at C-2. The molecular weight, reduced by two atomic mass units (Table 1), was consistent with a replacement of fluorine by a hydroxyl group. For F-5, only one singlet was observed in the aromatic region of the NMR spectrum (Table 2): this was to be assigned to the proton at C-2. An increase in the molecular weight of 14 (Table 1), implying the replacement of fluorine by a hydroxyl group and the addition of one oxygen atom at position C-5, implicated the catechol-type congener F-5 (Figure 3). The identity of F-2 and F-5 was confirmed on the basis of their molecular formulae determined by HR-MS (data not shown).

**Table 2:** ^1^H-NMR characteristics of the aromatic protons of PRA and metabolites produced by *G. striatum*

		Chemical	Coupling
Compound	H assignment	shift δ	constant *J *_H, F_
		(ppm)	(Hz)
PRA	H-2	8.92	
	H-5	7.87	14.5
F-1	H-2	8.41	
	H-5	7.96	14.8
F-2	H-2	9.06	
	H-5	7.64	
F-5	H-2	8.81	
F-13	H-2	8.80	
	H-5	7.08	
	H-2'	8.81	

Identification of metabolite F-13, 6-[(*E/Z*)-1-cyano-2-hydroxyethenyl]-1-cyclopropyl-4-oxo-1,4-dihydro-3-pyridinecarboxylic acid (Figure 3), was based on (i) its exact mass, [M+H]^+ ^= 247.0719, equating to the molecular formula C_12_H_10_N_2_O_4_; (ii) three singlets in the aromatic region of the NMR spectrum, assigned to H-5, H-2 and H-2' (Table 2); (iii) NMR signals of all protons of the cyclopropyl moiety (data not shown); and (iv) specific peaks of absorbance of the CN group at 2189 and 2218 cm^-1 ^in the FT-IR spectrum. However, whether the hydroxyl group at C-2' was located in *cis *or *trans *position to the CN group remains to be elucidated.

The application of [pyrrolidinopiperdine-7-^14^C]PRA as substrate to be degraded facilitated the identification of a seventh major metabolite, F-10, the intact pyrrolidinopiperidine substituent. Its characterization proceeded exactly as has been described for the piperazine residue of enrofloxacin; see Figure 4C in Wetzstein et al. (1997). F-10 was identified by co-chromatography, employing a chemically synthesized standard (not depicted), and confirmed by determination of its molecular weight (Table 1).

It should be mentioned that the quantities of six additional metabolites of PRA were too small to permit comprehensive structure elucidation. Retention times and HR-MS analysis-based molecular formulae suggested the presence of (i) one additional congener each of PRA and F-1, most likely carrying a mono and a dihydroxylated amine substituent; (ii) F-2, to which one oxygen atom had been added but water eliminated; and (iii) F-9, in which fluorine was replaced by a hydroxyl group; a labile *ortho*-aminophenol-type metabolite resembling key metabolite 77 of enrofloxacin (see Figure 5 in Karl et al. [2006]).

## Discussion

The basic metabolic pathway of PRA, a new veterinary FQ antibacterial drug, in the brown rot fungus *G. striatum *was similar to schemes established for other FQs such as enrofloxacin, ciprofloxacin and moxifloxacin. Hydroxylated primary metabolites of PRA, each representing a different class of compounds (Figure 3), were generated by hydroxyl radical-based decarboxylation (F-1), defluorination (F-2) and elimination of CN (F-6). The definitive identification of a catechol-type FQ congener, compound F-5, carrying one hydroxyl group each at C-5 and C-6, is described here for the first time. This was facilitated by the CN substituent blocking C-8, in contrast to F-5 of enrofloxacin Wetzstein et al. 1997 or ciprofloxacin Wetzstein et al. 1999, for which hydroxylation of position C-5 was indistinguishable from hydroxylation of C-8. Degradation of the amine substituent is represented by F-9.

The identification of metabolite F-13, consisting of the cyclopropyl-substituted pyridone part and C-atoms 7 and 8 of PRA, now linked by a double bond and carrying a hydroxyl and the CN group, respectively, proved fungal cleavage of the aromatic FQ core for the first time. The presence of a conjugated CN group was verified by IR-spectroscopy. Being part of a mesomeric system, the CN group is likely to have stabilized metabolite F-13 sufficiently as to permit its isolation and structure elucidation. The most likely intermediates, connecting F-5 with F-13 (Figure 3), imply: (i) hydroxyl radical-based elimination of the intact amine moiety (F-10), a reaction already observed for enrofloxacin and ciprofloxacin Wetzstein et al. 1997; 1999; (ii) twofold oxidation of the resulting pyrogallol-type intermediate and the formation of a *cis,cis*-muconic acid-type analog; and (iii) spontaneous twofold decarboxylation, finally providing F-13. Metabolite F-13 adds a unique type of compound to the plethora of 137 known metabolites of enrofloxacin (including the ethylpiperazine residue and CO_2_) produced by basidiomycetous fungi Karl et al. 2006;Wetzstein et al. 2006.

Six major metabolites of enrofloxacin and, in particular, 8-OH-PRA (F-6) have been shown to essentially have lost antibacterial activity Wetzstein et al. 2009;Wetzstein and Hallenbach 2011. Most recent observations suggest that minimum inhibitory concentrations of 8-OH-PRA may have been slightly (less than twofold) overestimated, due to its instability at 37°C, resulting in a half-life of about 2 days (Wetzstein H-G, unpublished data). Regardless, FQ residues such as those described herein appear to be unlikely to pose a significant risk due to selection of resistance in agricultural soils Wetzstein et al. 2009;Baquero et al. 2011.

Recently, metabolites of norfloxacin hydroxylated at position C-6 and C-8 have been reported to be formed by *Microbacterium *spp. isolated from wastewater Kim et al. 2011. Moreover, from 8-OH-norfloxacin, C-8 may be eliminated by *Candida palmioleophila *LA-1 Kim et al., Abstr 111th Annu Gen Meet Am Soc Microbiol, abstr. Q-2943, 2011. The corresponding metabolite should be indicative of a mechanism of aromatic ring cleavage different from that believed to be observed in this study.

Metabolic inactivation of FQs in mammals predominantly comprises glucuronidation of the carboxyl group and sulfation of an appropriate secondary amine function, if present in the amine substituent. Furthermore, *N*-4'-dealkylation, formylation and oxide formation as well as partial degradation of the C-7 amine substituent have been observed. However, core-hydroxylated metabolites were not reported, as reviewed by Dalhoff and Bergan (1998). Another major mechanism of FQ inactivation is *N-*4'-acetylation, in case of enrofloxacin following *N*-4'-deethylation, as catalyzed by the Zygomycete *Mucor ramannianus *Parshikov et al. 2000. For *G. striatum*, only *O*-acetylated congeners have been observed, exemplified by metabolites 13 and 62 described by Karl et al. (2006). Chemically synthesized *N*-acetyl-PRA provides for MICs similar to those of 8-OH-PRA (Wetzstein H-G, unpublished observation). However, it is unknown, whether PRA could serve as a substrate for the bacterial enzymes yet described.

Most notable, acetylation of the piperazine residue of ciprofloxacin or norfloxacin by environmental strains of *Mycobacterium *[Bibr B1][Bibr B2] has not yet been found in clinical strains. Furthermore, a FQ-resistant strain of *E. coli*, isolated from sewage sludge, contained the aminoglycoside transacetylase gene *aac(6')-Ib-cr *and was capable of modifying ciprofloxacin by acetylation Jung et al. 2009. This activity was first observed to be a plasmid-encoded FQ-resistance factor in Gram-negative species Robicsek et al. 2006. *N*-acetylation as well as N-oxide formation Parshikov et al. 2000;Karl et al. 2006 eliminates the positive charge of the amine residue, present at physiological pH. The resulting FQ congener is negatively charged, thus drug accumulation into the cytoplasm may be restricted or even prevented.

Strangely enough, the apparently complex degradation scheme described for enrofloxacin Karl et al. 2006;Wetzstein et al. 2006 has to be considered a relatively simple example, compared to degradation patterns to be expected for PRA, moxifloxacin and any other FQ with a more complex amine substituent. Giving rise to the variety of constitutional isomers, the number of discernible H-atoms in the amine moiety, potentially to be replaced by a hydroxyl group, amounts to four for enrofloxacin, but already to twelve for the pyrrolidinopiperidine residue of PRA; this structural feature extends to metabolites carrying a combination of a hydroxyl and a keto group or even a cleaved amine moiety. Hence, definitive structure elucidation of such metabolites would have to be based on isolated compounds and required a formidable analytical effort.

In independent studies assessing the chemical degradation of enrofloxacin, ciprofloxacin or other FQs, cleavage of the aromatic core could not yet be proved either, if based on metabolite identification. Work on ciprofloxacin confirmed several key metabolites observed with *G. striatum*: (i) F-1, F-2, F-6 and F-9 in a membrane anodic Fenton-type system (Xiao et al. [2010]; see also references 19, 22 and 25, therein); (ii) isatin and anthranilic acid-type metabolites formed during ozonation (Dewitte et al. 2008; and (iii) metabolites indicating degradation of the amine substituent by hydroxyl radicals, generated upon UV-irradiation of TiO_2 _Paul et al. 2007. Under the latter conditions, cleavage of the cyclopropyl moiety was observed as well. This leaves unexplored fungal degradation of the cyclopropyl-substituent, which, however, is a natural product found, e.g., in the lipids of *E. coli *Goldfine 1982. Two decades after the emergence of the notion of the non-biodegradability of FQs, briefly reviewed by Wetzstein et al. (2009), fungal degradation of all key structural elements of a FQ, in particular of the aromatic core, now has been proven.

## Competing interests

The authors are (HGW and JS) or have been (WK) employees of Bayer AG, as indicated.

## Authors' contributions

HGW carried out the microbiological experiments and drafted the manuscript. JS determined the optimal experimental conditions and performed chemical analyses. WK guided structure elucidation and provided all interpretations of the chemical raw data. All authors read and approved the final manuscript.
